# An intrusion detection model to detect zero-day attacks in unseen data using machine learning

**DOI:** 10.1371/journal.pone.0308469

**Published:** 2024-09-11

**Authors:** Zhen Dai, Lip Yee Por, Yen-Lin Chen, Jing Yang, Chin Soon Ku, Roohallah Alizadehsani, Paweł Pławiak

**Affiliations:** 1 Department of Computer System and Technology, Faculty of Computer Science and Information Technology, Universiti Malaya, Kuala Lumpur, Malaysia; 2 Department of Computer Science and Information Engineering, National Taipei University of Technology, Taipei Taiwan; 3 Department of Computer Science, Universiti Tunku Abdul Rahman, Kampar, Malaysia; 4 Institute for Intelligent Systems Research and Innovation (IISRI) Deakin University, Waurn Ponds, Australia; 5 Department of Computer Science, Faculty of Computer Science and Telecommunications, Cracow University of Technology, Warszawska, Krakow, Poland; 6 Institute of Theoretical and Applied Informatics, Polish Academy of Sciences, Bałtycka, Gliwice, Poland; Universiti Malaysia Sabah, MALAYSIA

## Abstract

In an era marked by pervasive digital connectivity, cybersecurity concerns have escalated. The rapid evolution of technology has led to a spectrum of cyber threats, including sophisticated zero-day attacks. This research addresses the challenge of existing intrusion detection systems in identifying zero-day attacks using the CIC-MalMem-2022 dataset and autoencoders for anomaly detection. The trained autoencoder is integrated with XGBoost and Random Forest, resulting in the models XGBoost-AE and Random Forest-AE. The study demonstrates that incorporating an anomaly detector into traditional models significantly enhances performance. The Random Forest-AE model achieved 100% accuracy, precision, recall, F1 score, and Matthews Correlation Coefficient (MCC), outperforming the methods proposed by Balasubramanian et al., Khan, Mezina et al., Smith et al., and Dener et al. When tested on unseen data, the Random Forest-AE model achieved an accuracy of 99.9892%, precision of 100%, recall of 99.9803%, F1 score of 99.9901%, and MCC of 99.8313%. This research highlights the effectiveness of the proposed model in maintaining high accuracy even with previously unseen data.

## Introduction

In today’s digitally connected world, cybersecurity has emerged as a critical concern for individuals, organizations, and governments. The rapid advancement of technology and the widespread adoption of the Internet have led to a surge in cyber threats, ranging from traditional malware and phishing attacks to more sophisticated and elusive tactics. Among these advanced threats, zero-day attacks are particularly insidious [1]. These attacks exploit undiscovered vulnerabilities in software or hardware, allowing threat actors to compromise systems without the prior knowledge of developers or defenders.

The integration of machine learning (ML) and deep learning (DL) has emerged as a promising approach to counter such threats. Unlike traditional rule-based systems, ML and DL models leverage large datasets of historical cyber events and network behavior to detect cyber threats more effectively [2, 3]. These models are capable of identifying known attack signatures and distinguishing novel attack patterns, thereby identifying emerging cyber risks and vulnerabilities [4].

This research introduces a novel intrusion detection model designed to enhance the detection of zero-day attacks in unseen data. The significant contributions of this research can be summarized as follows:

- Novel Approach: This study introduces an innovative intrusion detection model that combines autoencoders with Random Forest and XGBoost to detect cyberattacks, particularly focusing on previously unseen data.
- Improved Detection Performance: The proposed models, Random Forest-AE and XGBoost-AE, exhibit high accuracy even when confronted with previously unseen data. By leveraging autoencoders to capture intrinsic features during training, these models achieve robust performance in real-world intrusion detection scenarios.
- Comparative Analysis: A thorough comparison with current methods shows that the proposed models, especially Random Forest-AE, are better at finding cyber threats that haven’t been seen before.

The remainder of this paper is organized as follows: Section 2 reviews relevant literature; Section 3 details the research methodology and describes the proposed method configurations; Section 4 presents and discusses results using the publicly available CIC-MalMem-2022 dataset, demonstrating the superiority and reproducibility of the proposed method. Section 5 presents the challenges and solutions of this study and suggests directions for future research. Finally, Section 6 concludes by summarizing the research’s strengths and weaknesses.

## Related work

Detecting zero-day attacks is a critical challenge in cybersecurity, prompting extensive research into various methodologies. This section surveys existing solutions, positions the proposed work within the broader context of these approaches, and identifies gaps that it aims to address.

### Supervised learning techniques

Smith et al. conducted a study comparing the performance of supervised and unsupervised learning techniques on the Malware-Exploratory and CIC-MalMem-2022 datasets [5]. They used three clustering algorithms (K-Means, DBSCAN, and GMM) and seven classification algorithms (Decision Trees, Random Forests, AdaBoost, KNeighbors, Stochastic Gradient Descent, Extra Trees, and Gaussian Naïve Bayes). The study found high accuracy rates (over 90%) and consistent clustering outcomes, irrespective of feature correlation. While effective, these supervised techniques often struggle with zero-day attacks due to their reliance on known attack signatures.

### Deep learning models

Dener and Orman proposed a model using deep learning and machine learning methods for clustering and detecting malware in in-memory data. Their study on the balanced CIC-MalMem-2022 dataset using Pyspark on the Apache Spark big data platform compared nine algorithms [6], including Random Forest, Decision Tree, Gradient Boosting Tree, Logistic Regression, Naive Bayes, Linear Vector Supported Machine, Multilayer Perceptron, Deep Feedforward Neural Network, and Long Short-Term Memory [7, 8]. Logistic regression achieved the highest accuracy of 99.97%, closely followed by gradient-boosting trees with 99.94% accuracy. These models show promise but often require extensive computational resources and large datasets for training.

### Semi-supervised and unsupervised approaches

Mbona and Eloff introduced a method for detecting zero-day network intrusion attacks using semi-supervised machine learning and Benford’s law to identify crucial features distinguishing benign and malicious traffic [9]. They applied Gaussian mixture models (GMM) [10] and one-class support vector machines (OCSVM) [11] using the CICDDoS2019 [12], IOTIntrusion2020, and CIRA-CIC-DoHBrw-2020 [13] datasets. This approach helps address the challenge of limited labeled data but may still struggle with highly obfuscated attacks.

### Convolutional neural networks

Mezina and Burget proposed an expansive convolutional network for detecting and classifying obfuscated malware in memory. Using a dilated CNN model for multi-class classification of malware families on the CIC-MalMem-2022 dataset, they reported high accuracy rates for binary classification, particularly with Random Forest (0.99992), KNN (0.99966), and Decision Tree (0.99923) [3]. While CNNs are powerful, their complexity and need for substantial training data are potential drawbacks.

### Open-set identification

Soltani et al. introduced a framework for detecting and adapting to novel attacks in network traffic using deep learning and open-set identification methods. This framework addresses the challenge of zero-day attacks by incorporating open-set identification and handling classes not seen during training. Evaluated using the CIC-IDS2017 and CSE-CIC-IDS2018 [14] datasets, the framework effectively detected and adapted to zero-day attacks with an average accuracy of 99% [15]. This method shows potential but may require further validation on diverse datasets.

### Generative Adversarial Networks (GANs)

De Araujo-Filho et al. proposed an innovative unsupervised intrusion detection system (IDS) for 5G networks using GANs, temporal convolutional networks (TCNs), and self-awareness to detect known and zero-day attacks without labeled data. Their IDS, evaluated with the CICDDoS2019 dataset, achieved higher detection rates (0.9993) and shorter detection times compared to baseline GAN-based IDSs like FID-GAN and ALAD [2]. GANs offer a robust solution but can be challenging to train and stabilize.

### Feature engineering and pre-processing

Balasubramanian et al. emphasized the importance of pre-processing and feature engineering for memory-based malware detection. Using feature selection algorithms such as correlation heat maps, extra tree classifiers [16], and analysis of variance (ANOVA) [17], they evaluated the CIC-MalMem-2022 dataset. For binary classification, they reported high detection accuracy for Decision Tree (0.9999), Random Forest (0.9999), and SVM (0.9994) [18]. Effective pre-processing is crucial but can be time-consuming and computationally intensive.

### Gaps

Despite the advancements in these methodologies, significant challenges remain in detecting zero-day attacks. Existing solutions often rely heavily on large, labeled datasets or complex models that require extensive computational resources. Moreover, many approaches struggle to generalize well to unseen data, a critical requirement for effective zero-day attack detection.

The proposed research addresses these gaps by integrating an autoencoder-based anomaly detector with supervised learning algorithms (Random Forest and XGBoost). This novel approach enhances the detection of zero-day attacks by learning the characteristics of normal traffic and identifying deviations, thus improving the models’ ability to handle previously unseen data.

## Methodology

In this study, "unseen data" refers to data that the model has not encountered during the training phase. This concept is crucial for assessing the model’s ability to detect zero-day attacks, which are characterized by their novel and previously unknown nature. Unseen data can be categorized into two types:

1. Novel Attacks: These are entirely new types of attacks that exploit vulnerabilities not previously identified or recorded. Novel attacks represent a significant challenge for detection systems because they differ fundamentally from known attack patterns.
2. Variations of Known Attacks: These include slight modifications or variations of known attack patterns. While these are different from the specific instances used during training, they share underlying characteristics with known attacks.

**Fig 1:**
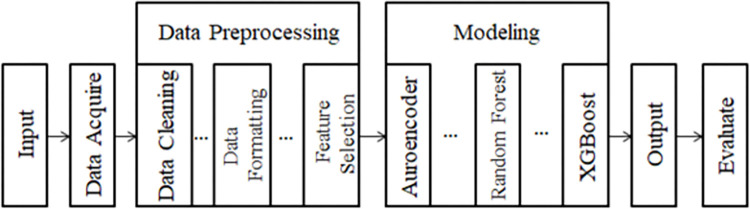
Flowchart of the proposed method.

The proposed method of the study is a hybrid approach combining a simple random forest and XGBoost with an autoencoder to address the performance degradation of the model when detecting unseen data (see Fig 1).

The proposed method includes data preprocessing, such as data cleaning, formatting, and feature extraction, in the data preprocessing stage, aimed at enhancing dataset quality and improving model generalization. In the modeling stage, the normal data is separated in advance from CIC-MalMem-2022. This enables the autoencoder to learn and memorize the features of normal data. An anomaly detector is then established by setting a threshold based on reconstruction errors. Subsequently, the classifier combines random forest and XGBoost models for classification. In the final stage, common metrics are used to evaluate the performance of the model to ensure its effectiveness in detecting and classifying unseen data.

### Data acquire

The dataset employed for this research is CIC-MalMem-2022, sourced from the Canadian Institute for Cyber Security (https://www.unb.ca/cic/datasets/malmem-2022.html). The CIC-MalMem-2022 dataset is one of the most recent and comprehensive datasets available for malware detection. It includes obfuscated malware samples, which are representative of real-world cyber threats such as spyware, ransomware, and Trojan malware. The dataset contains 57 features, providing a rich set of attributes for machine learning models to learn from. The dataset has 58,596 records, of which 29,298 are benign and 29,298 are malicious. This diversity in features allows for robust training and evaluation of the proposed models. Using a well-known public dataset like CIC-MalMem-2022 allows for direct comparison with other studies in the field. This helps in benchmarking the proposed models against existing solutions, highlighting improvements and innovations. Table 1 below shows that the dataset contains 57 features, of which two are categorical and the remaining 55 are numerical.

**Table 1:** Different features of the dataset.

Feature Type	No.	Feature Name	Data Type
Label	1.	Category	Categorical
Process Information	2.	pslist.nproc	Numerical
	3.	pslist.nppid	Numerical
	4.	pslist.avg_threads	Numerical
	5.	pslist.nprocs64bit	Numerical
	6.	pslist.avg_handlers	Numerical
DLL Information	7.	dlllist.ndlls	Numerical
	8.	dlllist.avg_dlls_per_proc	Numerical
Handles Information	9.	handles.nhandles	Numerical
	10.	handles.avg_handles_per_proc	Numerical
	11.	handles.nport	Numerical
	12.	handles.nfile	Numerical
	13.	handles.nevent	Numerical
	14.	handles.ndesktop	Numerical
	15.	handles.nkey	Numerical
	16.	handles.nthread	Numerical
	17.	handles.ndirectory	Numerical
	18.	handles.nsemaphore	Numerical
	19.	handles.ntimer	Numerical
	20.	handles.nsection	Numerical
	21.	handles.nmutant	Numerical
Loader Modules Information	22	ldrmodules.not_in_load	Numerical
	23	ldrmodules.not_in_init	Numerical
	24	ldrmodules.not_in_mem	Numerical
	25	ldrmodules.not_in_load_avg	Numerical
	26	ldrmodules.not_in_init_avg	Numerical
	27	ldrmodules.not_in_mem_avg	Numerical
Memory Analysis	28	malfind.ninjections	Numerical
	29	malfind.commitCharge	Numerical
	30	malfind.protection	Numerical
	31	malfind.uniqueInjections	Numerical
Psxview Information	32	psxview.not_in_pslist	Numerical
	33	psxview.not_in_eprocess_pool	Numerical
	34	psxview.not_in_ethread_pool	Numerical
	35	psxview.not_in_pspcid_list	Numerical
	36	psxview.not_in_csrss_handles	Numerical
	37	psxview.not_in_session	Numerical
	38	psxview.not_in_deskthrd	Numerical
	39	psxview.not_in_pslist_false_avg	Numerical
	40	psxview.not_in_eprocess_pool_false_avg	Numerical
	41	psxview.not_in_ethread_pool_false_avg	Numerical
	42	psxview.not_in_pspcid_list_false_avg	Numerical
	43	psxview.not_in_csrss_handles_false_avg	Numerical
	44	psxview.not_in_session_false_avg	Numerical
	45	psxview.not_in_deskthrd_false_avg	Numerical
Module and Service Information	46	modules.nmodules	Numerical
	47	svcscan.nservices	Numerical
	48	svcscan.kernel_drivers	Numerical
	49	svcscan.fs_drivers	Numerical
	50	svcscan.process_services	Numerical
	51	svcscan.shared_process_services	Numerical
	52	svcscan.interactive_process_services	Numerical
	53	svcscan.nactive	Numerical
Callback Information	54	callbacks.ncallbacks	Numerical
	55	callbacks.nanonymous	Numerical
	56	callbacks.ngeneric	Numerical
Label	57	Class	Categorical

However, we do acknowledge that while the CIC-MalMem-2022 dataset is comprehensive, it represents a specific snapshot of malware threats. Future work should include evaluations of additional datasets to validate the generalizability of the proposed models. Moreover, implementing cross-dataset validation, which involves training the models on one dataset and testing them on another, will also be considered in the future because this approach might provide insights into how well the models generalize across different types of data and attack patterns.

### Data preprocessing

Following dataset acquisition, preprocessing steps are essential to enhance the effectiveness of machine learning model recognition and information extraction. Data quality significantly influences model training. The preprocessing techniques adopted in this study encompass data cleaning, feature selection, and data formatting.

### Data cleaning

Data cleaning is pivotal to eliminating outliers, duplicates, missing values, and noisy data from the dataset. This process enhances machine learning performance by removing irrelevant and noisy information.

### Data formatting

Data formatting is the process of organizing and structuring data in a standardized way to facilitate its storage, retrieval, and analysis. It involves converting raw data into a standardized format, following predefined rules or conventions. This ensures the consistency, reliability, and compatibility of the algorithm at runtime.

### Feature selection

Feature selection aids in choosing the most pertinent and informative features for analysis, diminishing dimensionality, and augmenting model efficiency. It guards against overfitting, thereby boosting model performance. In this research, the feature selection is conducted with Scikit-learn, a third-party machine learning library based on Python.

### Modeling

In the modeling phase, anomaly detection is initially conducted using an autoencoder (AE) [19]. AE is composed of an encoder and a decoder. The encoder compresses the input data into low-dimensional representations, and the decoder is used to reconstruct the original input data. AE was originally an unsupervised learning model, but it has been modified to become a semi-supervised learning model to perform feature selection and anomaly detection in this study. Normal samples are used to train the autoencoder model.

By training the model with normal data samples, the anomaly detector learns which behavioral features are normal before it can filter out the abnormal samples.

After filtering out the abnormal samples, two supervised machine learning models, random forest [20] and XGBoost [21], are used to classify the attack categories. The random forest model is an integrated learning technique that merges multiple decision trees to enhance predictive performance and counteract overfitting. Random forest can improve accuracy and stability in a variety of classification and regression scenarios [4]. The algorithm is particularly adept at handling high-dimensional datasets by creating a large number of decision trees, each trained on a random subset of the data.

XGBoost is an ensemble learning method that combines multiple weak learners (usually decision trees) to improve accuracy and reduce overfitting [22]. Through collaborative decision-making, XGBoost improves accuracy and consistency in a variety of classification and regression tasks.

Finally, optimization is conducted by adjusting hyperparameters to further improve the model’s performance.

### Autoencoder

**Fig 2:**
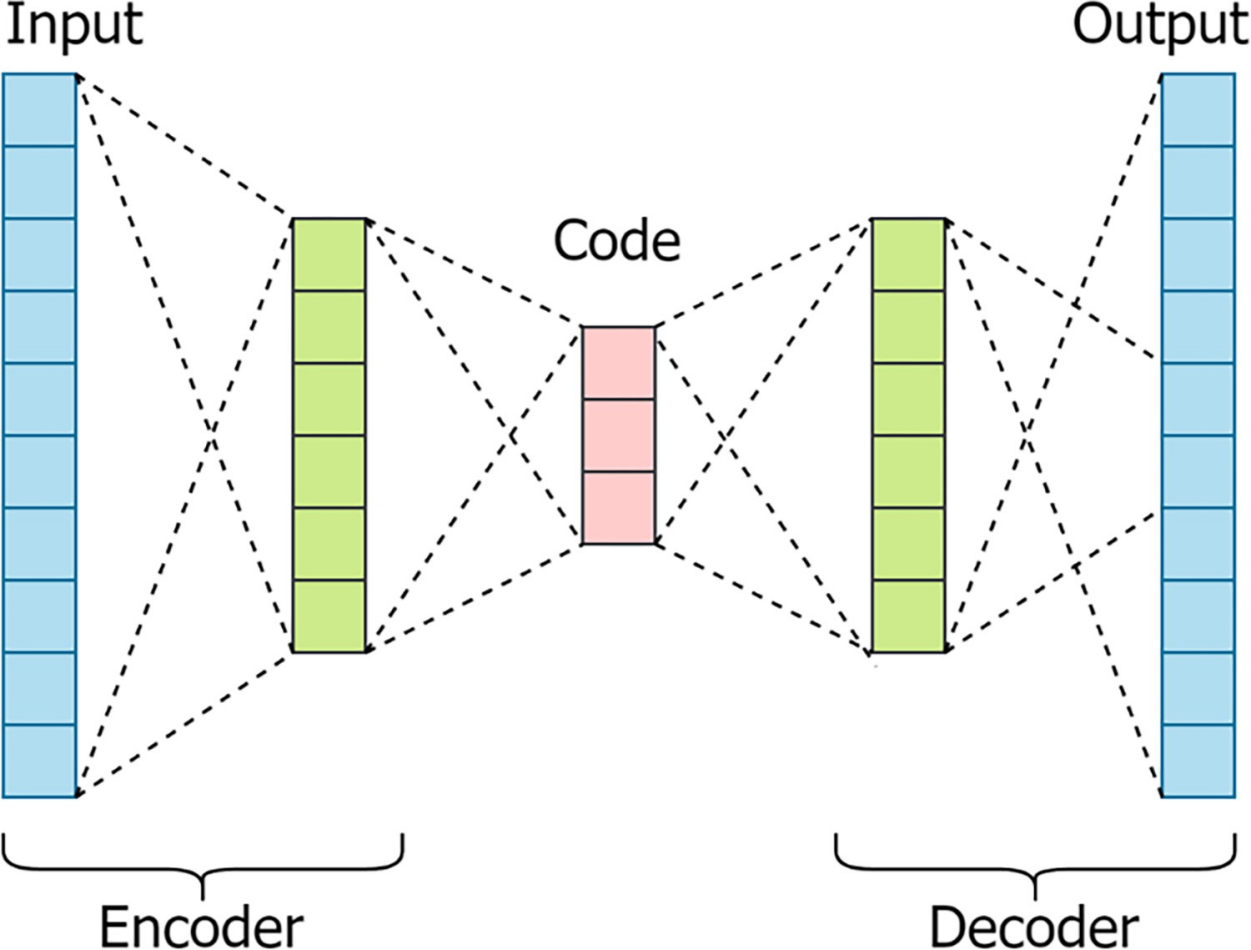
Schematic representation of the autoencoder.

An autoencoder is composed of an encoder and a decoder, with the objective of learning the compression features of the data input. The autoencoder formulation can be divided into two main parts: the encoding function (encoder) and the decoding function (decoder). Fig 2 shows the processing flow of the autoencoder.

Encoder Function: The encoding function takes the input data and maps it to a lower-dimensional representation. The formula for the encoder can be represented as follows:

h=f(x) (1)

where:

*x*: Input Data.

*h*: Encoded representation.

*f*(): Encoding function, such as a dense layer. The rectified linear unit (ReLU) and sigmoid are used in this research.

Decoder Function: The decoding function reconstructs the original input data from the encoded representation. The formula for the decoder can be represented as follows:

$$\hat{x}=g(h)$$ (2)

where:

$\hat{x}$: Reconstructed data.

*h*: Encoded representation.

*g*(): Decoding function, also implemented as a neural network layer, is the mirror image of the encoding layer.

Objective Function (Loss Function): The training of an autoencoder involves minimizing a loss function, which measures the difference between the input data and the reconstructed data. Common loss functions include mean squared error (MSE) for continuous data or binary cross-entropy for binary data. The objective function can be represented as:

$$L(x,\hat{x})$$ (3)

where:

ℒ: Loss function.

*x*: Input data.

$\hat{x}$: Reconstructed data.

Overall Autoencoder Objective: The overall objective of training an autoencoder is to minimize the reconstruction error, i.e., the difference between the input data and the reconstructed data. This is achieved by adjusting the weights and biases of the neural network during the training process.

$$min_{\theta }\frac{1}{N}\Sigma_{\dot{l}=1}^{N}L(x_{i},g(f(x_{i})))$$ (4)

where:

*θ*: Parameters (weights and biases) of the autoencoder neural network.

*N*: Number of training samples.

$x_{\dot{l}}$: Individual training samples.

### Random forest

**Fig 3:**
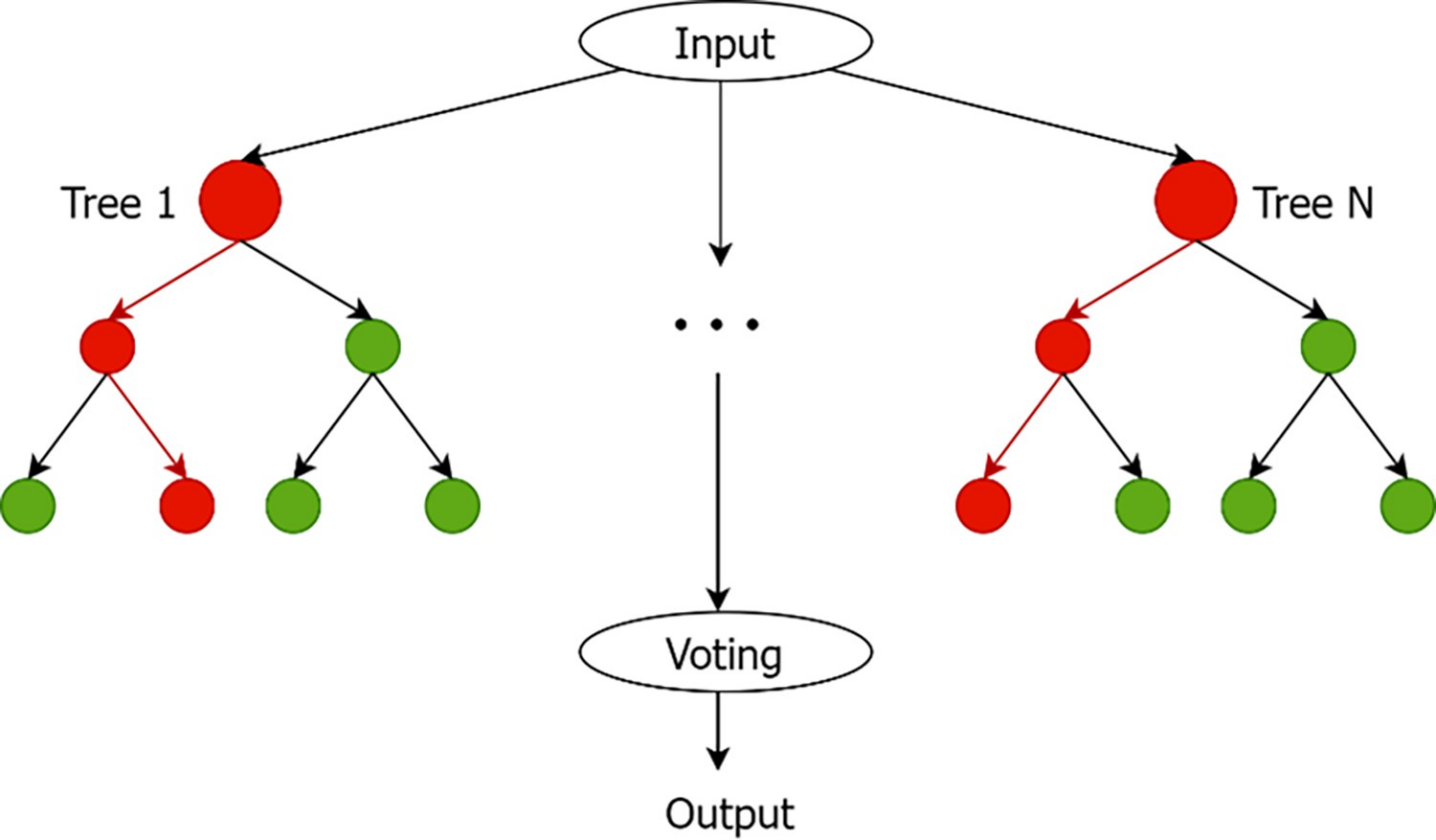
Schematic representation of a random forest.

Random forest is an ensemble learning technique that constructs multiple decision trees during training and outputs the mode of the classes (classification) or the mean prediction (regression) of the individual trees. The formula for Random Forest can be described in terms of decision trees and ensemble averaging. Fig 3 shows the processing flow of Random Forest.

Decision Tree: Random Forest is built upon decision trees; below is the basic formula for a decision tree.

$${\hat{y}}_{i}=T(x_{i})$$ (5)

where:

${\hat{y}}_{i}$: Predicted output for the i-th observation.

x_i_: Input features for the i-th observation.

T(): Decision tree model that maps input features to the predicted output.

Ensemble Averaging: Random Forest is a machine learning algorithm that combines the predictions from multiple decision trees to make a more robust and accurate prediction. The prediction ensemble is usually obtained by taking a majority vote (for classification) or averaging (for regression) of all individual tree predictions. This study is used for classification.

$$RF(x_{i})=\frac{1}{Ntrees}\Sigma_{\dot{j}=1}^{Ntrees}=1NtreesTj(xi)$$ (6)

where:

RF(x_i_): Random Forest prediction for the i-th observation.

*N*trees: The total number of trees in the Random Forest.

*Tj*(): Prediction from the j-th decision tree in the ensemble.

### XGBoost

**Fig 4:**
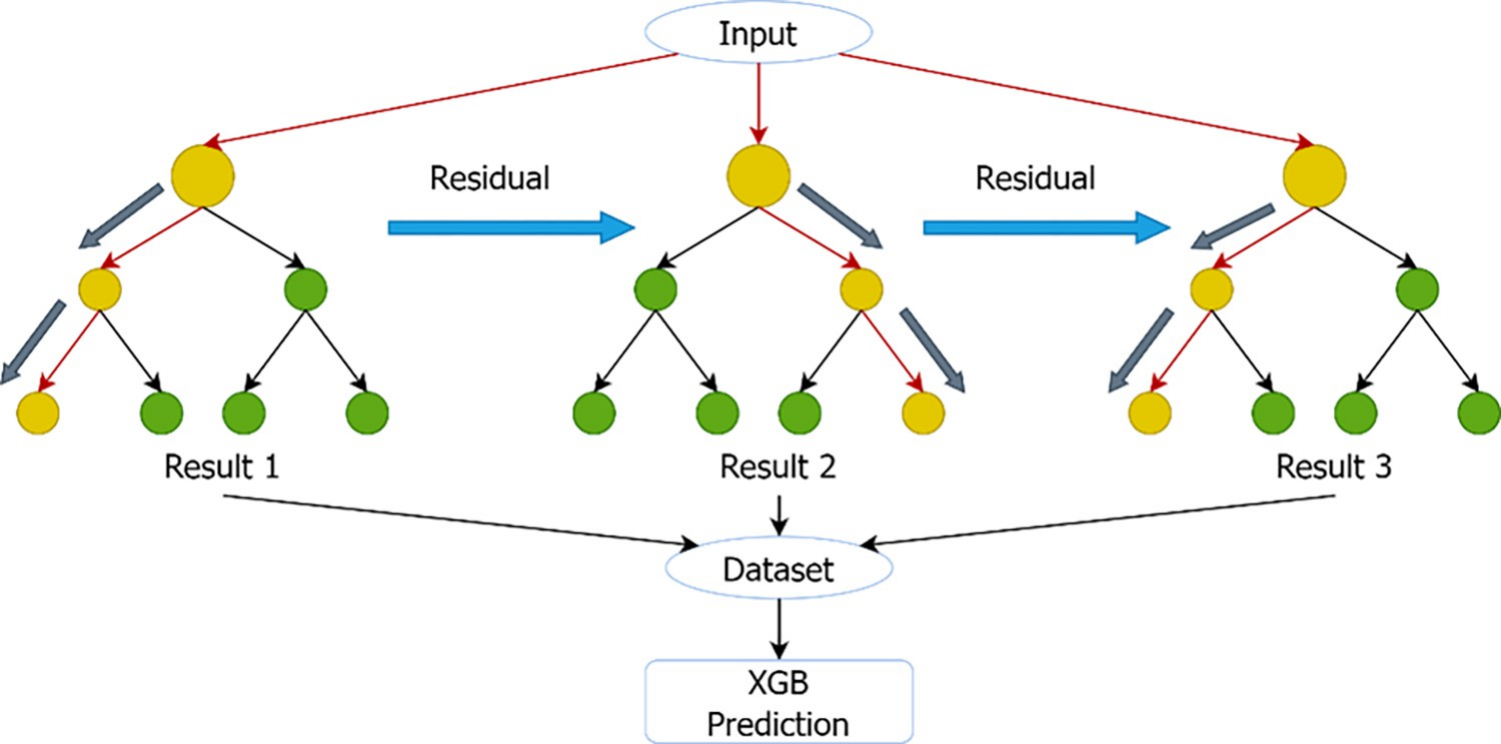
Schematic representation of XGBoost.

XGBoost (Extreme Gradient Boosting) [23] is a scalable and efficient implementation of the gradient boosting framework. The general formula for XGBoost can be described as an additive model, where each term corresponds to a weak learner, typically a decision tree, added to the ensemble. And Fig 4 shows the processing flow of XGBoost.

The objective is to minimize a regularized objective function that combines a loss term and a regularization term.

The formula for the XGBoost prediction.

$${\hat{y}}_{i}^{\left(t\right)}=\Sigma_{k=1}^{T}fk(x_{i}^{\left(t\right)})$$ (7)

where:

${\hat{y}}_{i}^{\left(t\right)}$: Predicted output for the i-th observation at iteration t.

T: The total number of weak learners (trees) in the ensemble.

$fk(x_{i}^{\left(t\right)})$: Prediction from the k-th weak learner at iteration t for the i-th observation.

### Random Forest-AE and XGBoost-AE

Step 1: Autoencoder Anomaly Detection Classification

The autoencoder reconstructs input data and classifies it as normal or abnormal based on the reconstructed error. In the training phase, it is trained on normal data, learning the characteristics of normal data and constructing a function that works only on normal data. The training process continuously carries out the reconstruction process, and the model’s weights are updated according to the reconstruction error to ensure the correct reconstruction of normal data. In practice, a threshold is set to determine the reconstruction error and classify the input data as normal or abnormal, and the label will be assigned to either normal or abnormal.


$$\varepsilon (X)=L(x,\hat{x})$$ (8)

Step 2: Secondary Training: Combining Reconstruction Errors and Labeling

Step 1 explains how the autoencoder worked in this study. By extending the Step 1 method, the normal and abnormal data, which have been fully classified and labeled, are merged into one dataset. This merged dataset is split into a training set and a test set and combined with the reconstruction error as input to the Random Forest and XGBoost classifiers. The classifiers are trained on the training set and evaluated on the test set. The effectiveness of the models will be tested using an unseen dataset generated from the CIC-MalMem-2022 dataset after completing all training.

### Evaluation

Several common performance metrics, such as the confusion matrix, accuracy, precision, recall, F1-score, and Matthews Correlation Coefficient (MCC), are used to evaluate the model’s performance. These metrics are described below. Confusion Matrix (see Table 2):

**Table 2:** Confusion matrix.

Actual	Positive	Negative
Positive	TP	FN
Negative	FP	TN

True Positive (TP): the number of positive samples correctly identified.

False Positive (FP): the number of false negative samples.

True Negative (TN): the number of negative samples that are correctly identified.

False Negative (FN): the number of positive samples that are underreported.

**Fig 5:**
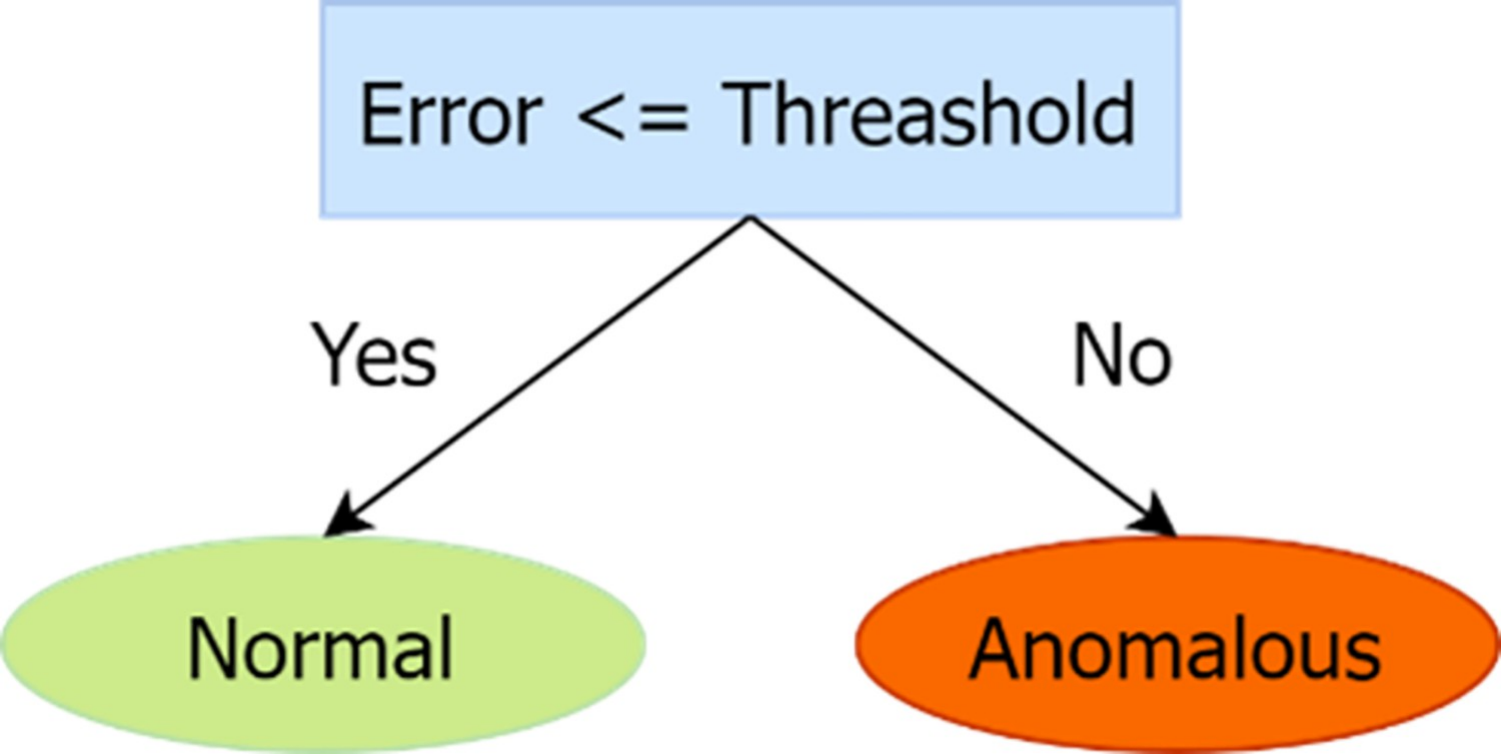
Schematic representation of a reconstructed error.

Fig 5 shows a schematic representation of the reconstructed error.

Accuracy: percentage of total samples with correct predictions.


$$Accuracy=\frac{TP+TN}{TP+FN+TN+FP}$$ (9)

Precision: proportion of correct predictions that are positive as a percentage of all positive predictions.


$$Precision=\frac{TP}{TP+FP}$$ (10)

Recall: Higher values of recall indicate better performance and a greater probability that anomalous samples will be judged to be anomalous.


$$Recall=\frac{TP}{TP+FN}$$ (11)

F1-Score: the ratio of the mean to the geometric mean; higher means better.


$$F1‐Score=2\times \frac{Recall\times Precision}{Recall+Precision}$$ (12)

Matthews Correlation Coefficient (MCC): A balanced measure for binary classifications, taking into account all four quadrants of the confusion matrix.


$$MCC=\frac{\left(TP\times TN)-(FP\times FN\right)}{\sqrt{\left(TP\times FP)(TP\times FN)(TN\times FP)(TN\times FN\right)}}$$ (13)

To achieve the main goal of this study, the model must attain a high level of accuracy in detecting unseen data. The test set will comprise 20% of the IDS dataset CIC-MalMem-2022, serving as a subset test set. Additionally, a new dataset will be generated based on the sampling of CIC-MalMem-2022 to represent unseen data, thus demonstrating the validity of the study. This new dataset will possess the same features as the training set, ensuring that the test set maintains identical dimensions and features. This precaution prevents the model from making incorrect classifications due to differences in features and dimensions. Incorporating the dataset sampled from CIC-MalMem-2022 into the test set streamlines the data preprocessing process.

We have conducted a detailed study comparing different train-test splits for our proposed methods (RandomForest-AE and XGBoost-AE). The experimental results, presented in Table 3, illustrate the performance across various metrics, providing a comprehensive view of the models’ robustness and generalizability. The 80/20 train-test split produced the best results for our proposed methods. Therefore, in this study, we use an 80/20 train-test split.

**Table 3:** Comparison of different train-test splits.

Metric	XGBoost-AE (80/20)	RandomForest-AE (80/20)	XGBoost-AE (70/30)	RandomForest-AE (70/30)	XGBoost-AE (60/40)	RandomForest-AE (60/40)
Accuracy	99.96%	99.99%	99.94%	99.96%	99.92%	99.94%
Precision	99.98%	100%	99.95%	99.97%	99.93%	99.96%
Recall	99.96%	99.98%	99.93%	99.95%	99.91%	99.94%
F1-Score	99.97%	99.99%	99.94%	99.96%	99.92%	99.95%
MCC	99.96%	99.99%	99.92%	99.94%	99.90%	99.93%

### Methodology for ensuring truly novel attacks in unseen data

1. **Separate Test Set:** The unseen data used for evaluation is drawn from a portion of the CIC-MalMem-2022 dataset that was not included in the training set. This ensures that the data used for testing the model’s performance is genuinely new to the model.
2. **Generation of Unseen Data:** To simulate novel attacks, a separate subset of the dataset was held out during the training phase. This subset includes diverse and complex attack scenarios to mimic real-world conditions where novel attacks may arise. This approach ensures that the model is tested against data it has not been trained on.
3. **Anomaly Detection:** The autoencoder-based anomaly detection component is crucial for identifying truly novel attacks. By learning the characteristics of normal traffic, the autoencoder can detect deviations that indicate novel attacks. This method helps in distinguishing novel attacks from mere variations of known attacks.

### Experiment setting

**Fig 6:**
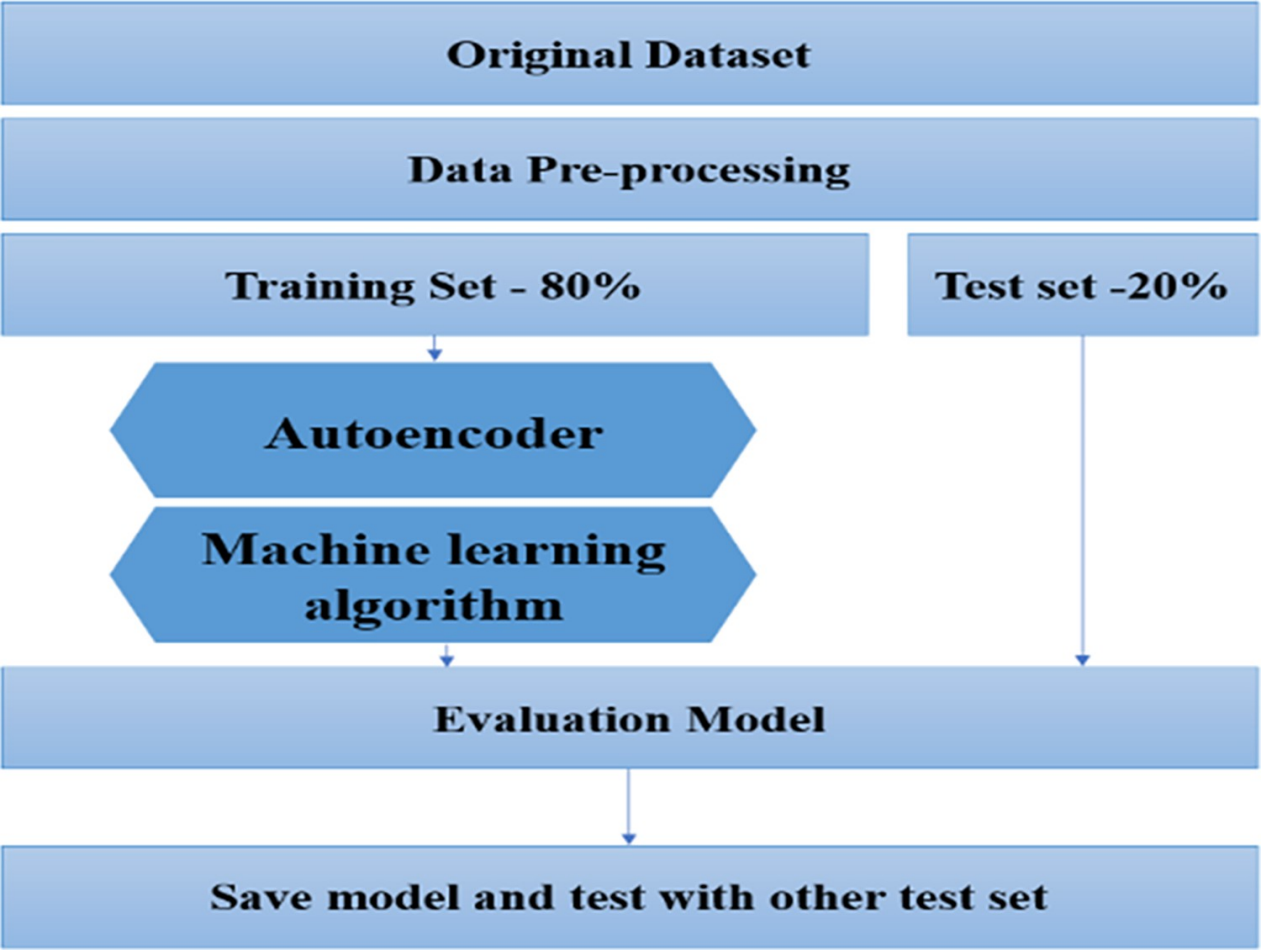
Experiment flow structure.

The study involved the data processing and modeling of machine learning algorithms on the Google collaborative platform using the Python programming language and libraries such as Pandas, NumPy, Sklearn, and Keras. Fig 6 illustrates the experiment flow structure used in this study. The steps are illustrated as follows:

1. **Data Loading and Initial Inspection:** The dataset was loaded into a Pandas DataFrame for manipulation and analysis. The initial inspection involved checking for null values, duplicates, and an overview of the dataset structure. The following is the Phyton coding for loading the dataset into a Pandas DataFrame:
   import pandas as pd
   df = pd.read_csv(’path_to_CIC_MalMem_2022_dataset.csv’)
   print(df.info())
2. **Data Cleaning:** Data cleaning involved removing null values, duplicates, and irrelevant data. Outliers and noisy data were also handled to improve the model’s performance. This process enhances the dataset’s quality, ensuring that the model is trained on accurate and relevant information. The following is the Phyton coding for data cleaning:
   # Remove duplicates
   df.drop_duplicates(inplace = True)
   # Handle missing values
   df.fillna(method = ’ffill’, inplace = True)
3. **Data Formatting:** The dataset includes 57 features, with 55 being numerical and 2 categorical. Categorical features were converted to numerical values using one-hot encoding. This transformation ensures that all features are in a numerical format suitable for machine learning algorithms. The following is the Phyton coding for one-hot encoding of categorical features:
   # One-hot encoding of categorical features
   df = pd.get_dummies(df, columns = [’categorical_feature_1’, ’categorical_feature_2’])
4. **Feature Selection:** Feature selection was performed to identify the most relevant features for the model, reducing dimensionality and enhancing performance. The Scikit-Learn library was used for this purpose. This step helps in selecting the most informative features, guarding against overfitting, and improving model efficiency. The following is the Phyton coding for feature selection:
   from sklearn.feature_selection import SelectKBest, f_classif
   X = df.drop(columns = [’target’])
   y = df[’target’]
   selector = SelectKBest(f_classif, k = 30) # Select the top 30 features
   X_new = selector.fit_transform(X, y)
5. **Data Splitting:** The dataset was split into training and testing sets, with 80% of the data used for training and 20% for testing. A seed value was set to ensure reproducibility. This split ensures that the model’s performance can be evaluated on an independent subset, helping to identify potential overfitting or underfitting issues. The following is the Phyton coding for data splitting:
   from sklearn.model_selection import train_test_split
   X_train, X_test, y_train, y_test = train_test_split(X_new, y, test_size = 0.2, random_state = 42)
6. **Autoencoder Architecture and Hyperparameter Selection**: The autoencoder used in this study serves as an anomaly detector. It was trained exclusively on benign data to learn the characteristics of normal traffic. The architecture and hyperparameters of the autoencoder were carefully selected to optimize its performance.
   Architecture
   The autoencoder consists of an input layer, a hidden layer with 32 neurons, and an output layer. The hidden layer uses the Rectified Linear Unit (ReLU) activation function, while the output layer uses the Sigmoid activation function (see the following for the Phyton coding implementation).
   from keras.models import Sequential
   from keras.layers import Dense
   autoencoder = Sequential()
   # Input layer
   autoencoder.add(Dense(input_dim = 30, units = 30, activation = ’relu’))
   # Hidden layer
   autoencoder.add(Dense(units = 32, activation = ’relu’))
   # Output layer
   autoencoder.add(Dense(units = 30, activation = ’sigmoid’))
   Hyperparameter Selection
   The autoencoder was compiled using the Adam optimizer and mean squared error (MSE) as the loss function. The model was trained for 100 epochs, which was found to be optimal after comparing performance with 200, 500, and 1000 epochs. Training for 100 epochs provided similar results in a shorter period, which is beneficial for re-training to handle evolving cyberattacks (see the following for the Phyton coding implementation).
   autoencoder.compile(optimizer = ’adam’, loss = ’mean_squared_error’)
   autoencoder.fit(X_train, X_train, epochs = 100, batch_size = 32, validation_split = 0.2)
7. **XGBoost-AE and Random Forest-AE**: The XGBoost-AE and Random Forest-AE models combine the anomaly detection capability of the autoencoder with traditional supervised learning algorithms. The reconstruction error from the autoencoder is used to classify the data as normal or abnormal. If the reconstruction error exceeds a set threshold, the data is classified as abnormal (see the following for the Phyton coding implementation).
   from xgboost import XGBClassifier
   from sklearn.ensemble import RandomForestClassifier
   # Training XGBoost-AE
   xgb_ae = XGBClassifier()
   xgb_ae.fit(X_train, y_train)
   # Training RandomForest-AE
   rf_ae = RandomForestClassifier()
   rf_ae.fit(X_train, y_train)
8. **Normalization:** Data normalization was applied to standardize the feature values, ensuring they contribute equally to the model’s performance. Standardization is crucial for models like Random Forest and XGBoost to perform optimally, especially when features have different scales. The following is the Phyton coding for normalization:
   from sklearn.preprocessing import StandardScaler
   scaler = StandardScaler()
   X_train = scaler.fit_transform(X_train)
   X_test = scaler.transform(X_test)

By meticulously preparing the data through these steps, the foundation is laid for reliable and effective machine learning models. This preparation is crucial, as it directly impacts the model’s ability to generalize and perform well on unseen data.

## Results and discussion

This paper evaluated four machine learning detection models: Random Forest, XGBoost, Random Forest-AE, and XGBoost-AE. The key focus was on assessing the effectiveness of the autoencoder-enhanced models (Random Forest-AE and XGBoost-AE) in detecting previously unseen data in intrusion detection. The results obtained from these models are compared to highlight their performance.

### XGBoost-AE and Random Forest-AE performance

The enhanced models, XGBoost-AE and Random Forest-AE, integrate an autoencoder for anomaly detection before applying the traditional Random Forest and XGBoost algorithms. This additional step aims to improve the models’ ability to detect previously unseen data.

**Fig 7:**
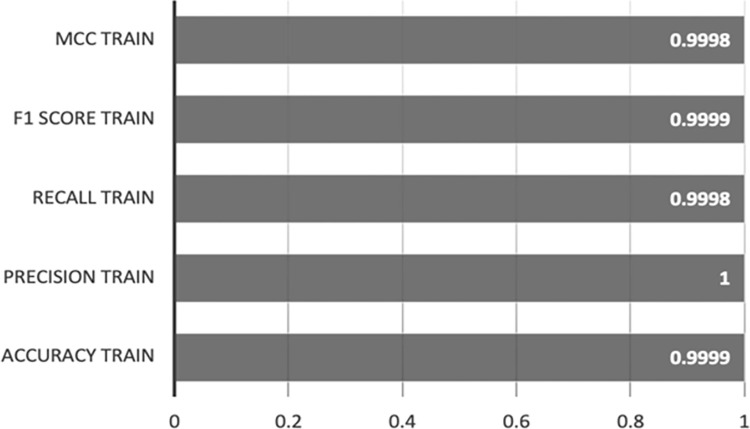
XGBoost-AE training set results.

Fig 7 illustrates the performance metrics for XGBoost-AE on the training set, showcasing accuracy, precision, recall, F1 scores, and MCC, all of which achieve scores between 0.9998 and 1.

**Fig 8:**
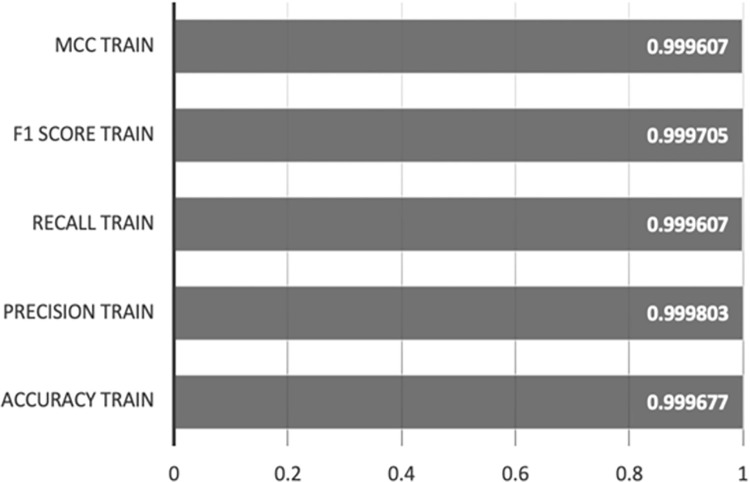
XGBoost-AE test set results.

The outcomes are depicted in Fig 8, showcasing the model’s performance on the test set, with an accuracy of 0.999677, a precision of 0.999803, a recall of 0.999607, an F1 score of 0.999705, and an MCC of 0.999607.

**Fig 9:**
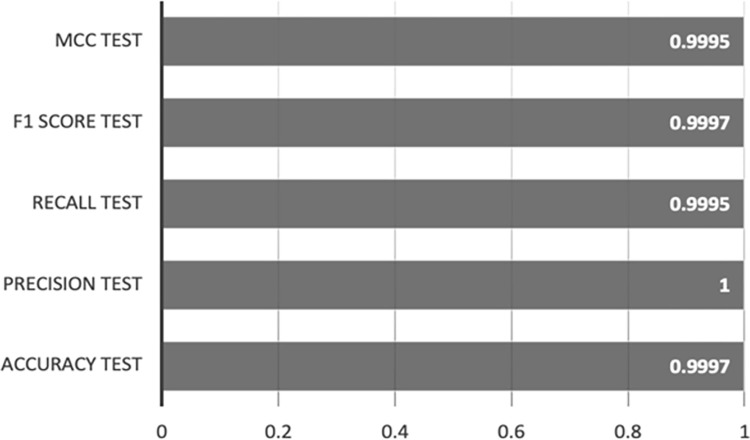
XGBoost-AE unseen dataset results.

Furthermore, the confusion matrix for the test set provides deeper insight, revealing 4195 true positives—instances that the model correctly identified as positives—and 1 false positive, indicating that the model incorrectly classified only 1 negative instance as positive. Additionally, there were 2 instances that were erroneously predicted as negative and 5092 true negatives—negative instances that were correctly identified. Fig 9 exhibits the test outcomes of XGBoost-AE on the unseen dataset.

The results underscore the XGBoost-AE model’s ability to sustain excellent performance on an unseen dataset. This comprehensive evaluation underscores the robustness and reliability of the XGBoost-AE model.

**Fig 10:**
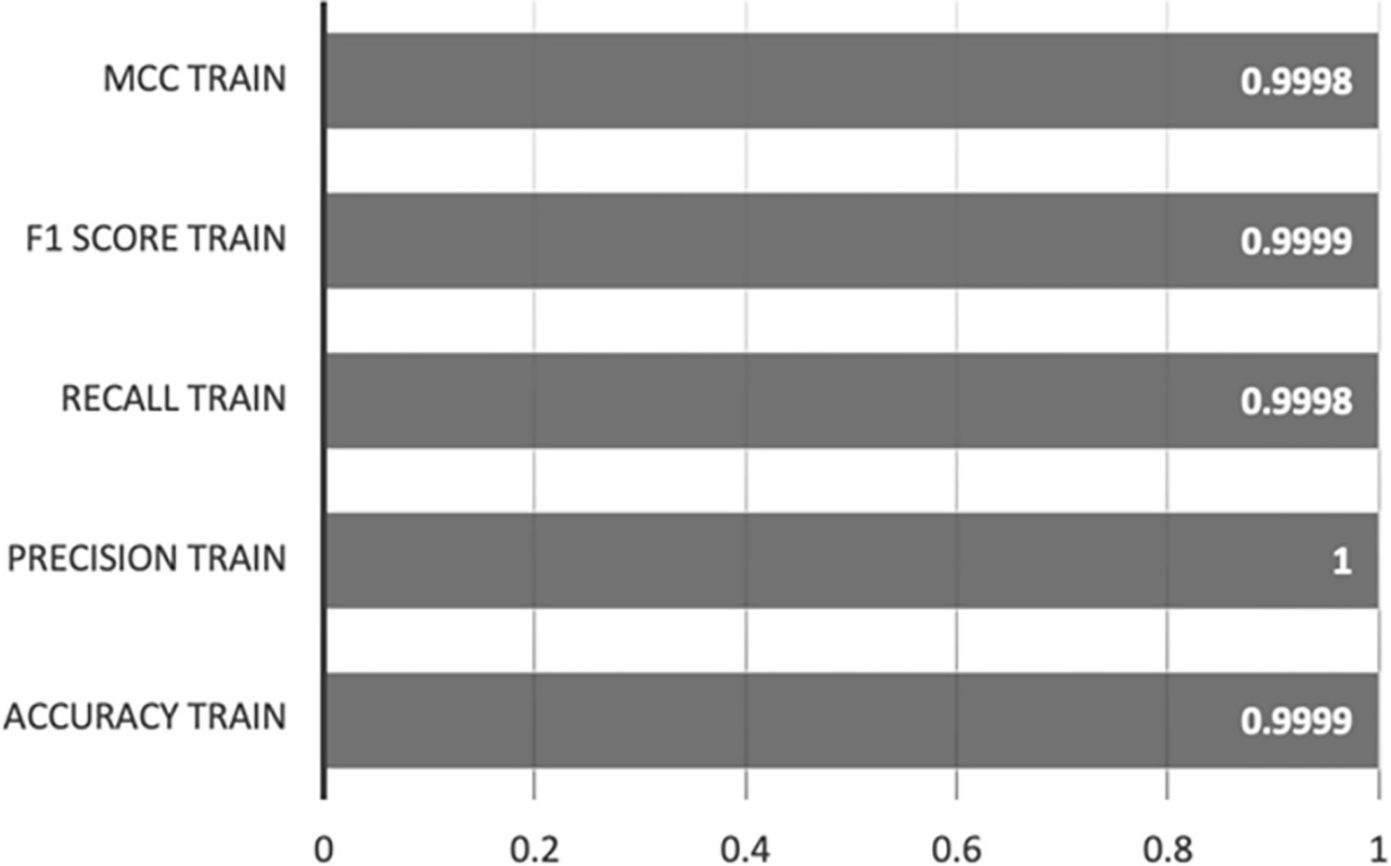
Random Forest-AE training set results.

**Fig 11:**
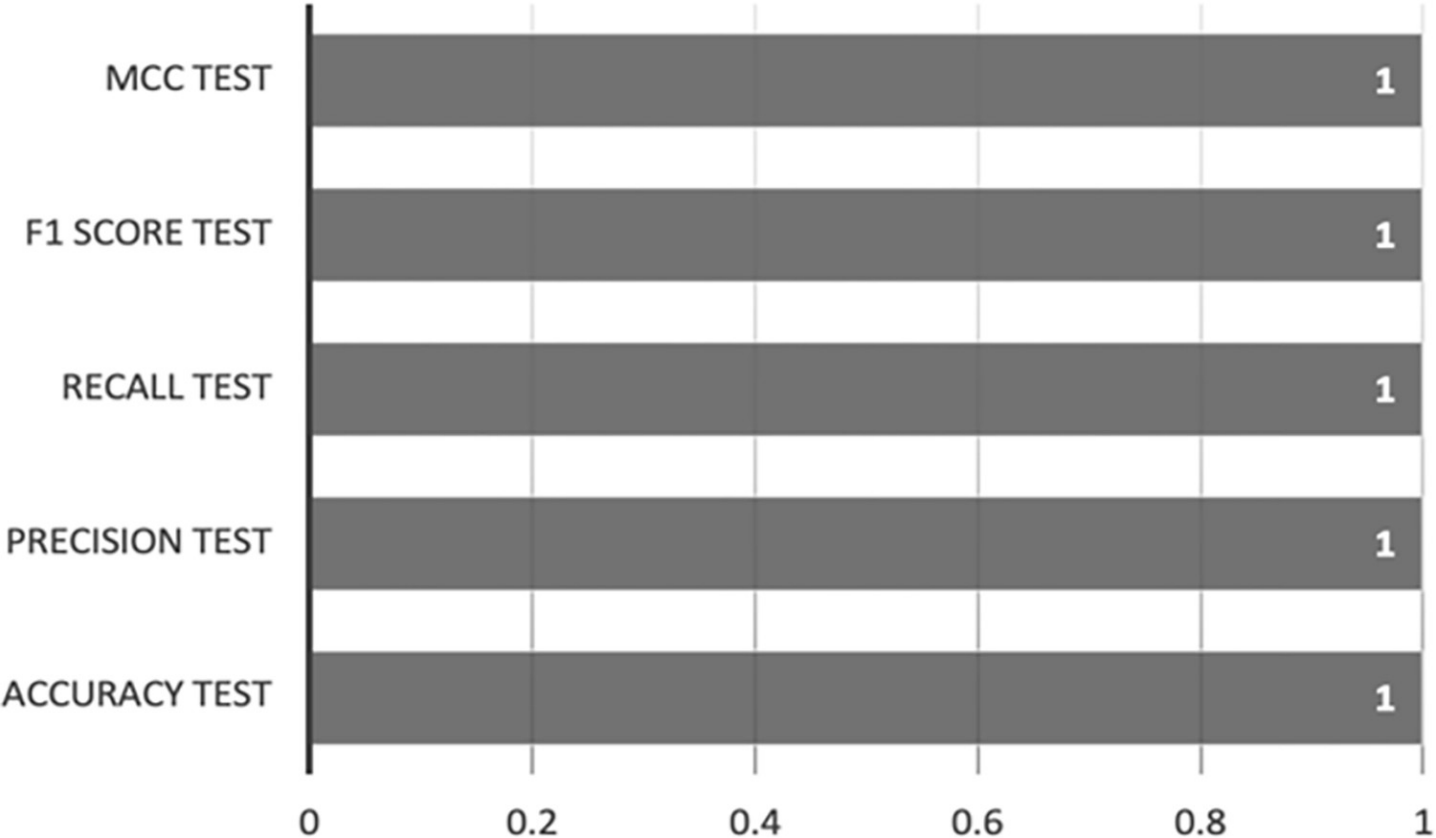
Random Forest-AE test set results.

Figs 10 and 11 exhibit the outcomes of implementing the Random Forest-AE method on both the training and test sets. The performance metrics acquired on the training set, encompassing accuracy, precision, recall, F1 score, and MCC, demonstrate optimal results, with all metrics achieving scores ranging from 0.9998 to 1. This alignment with the XGBoost-AE outcomes underscores the model’s robustness and reliability during the training phase.

**Fig 12:**
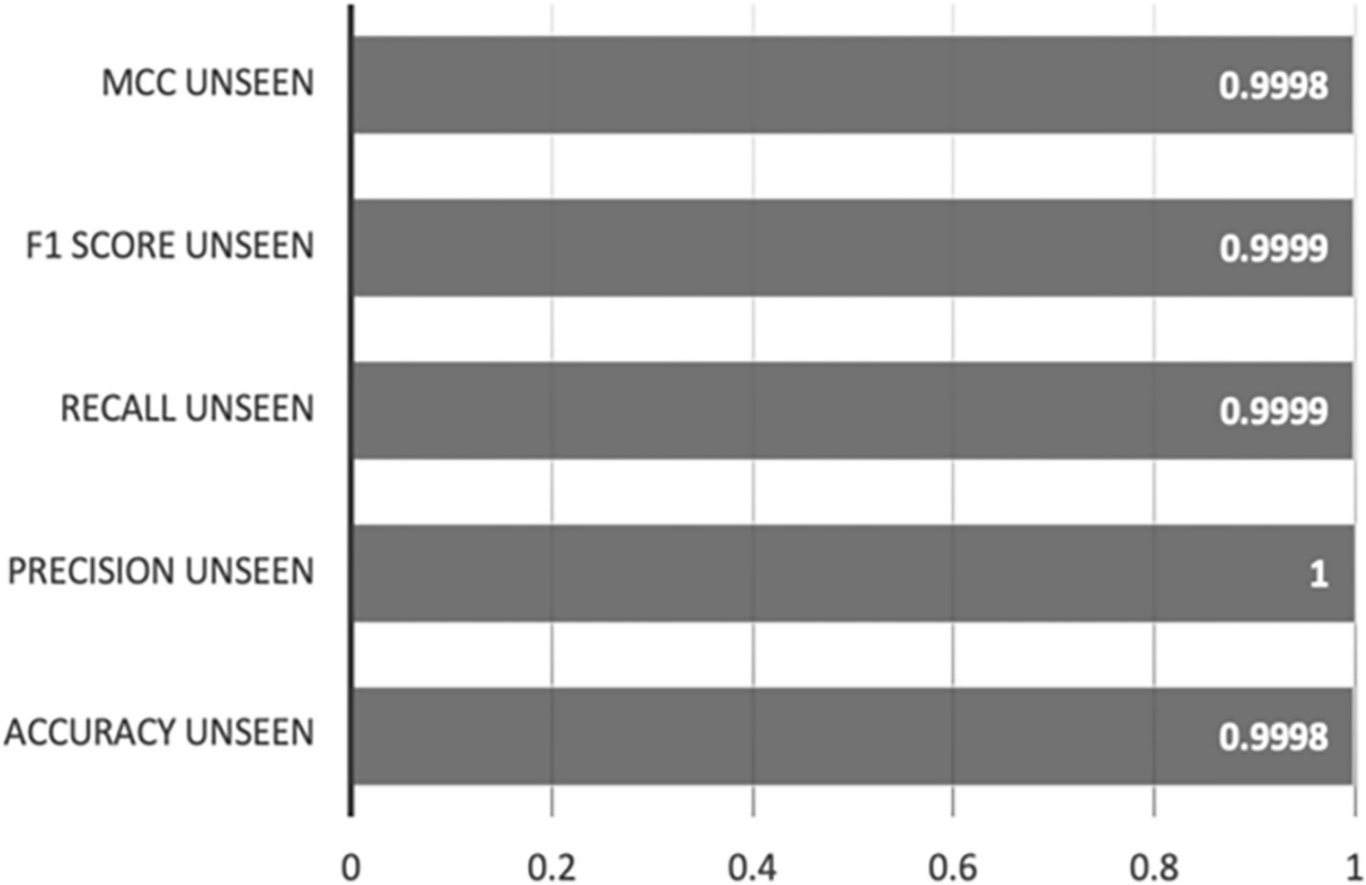
Random Forest-AE unseen dataset results.

Fig 12 showcases the test outcomes of the Random Forest-AE model on the unseen dataset. The model continues to perform exceptionally well, garnering near-perfect scores across all metrics, indicating that the model’s performance remains uncompromised by the unseen data.

### Comparative analysis with traditional models

**Fig 13:**
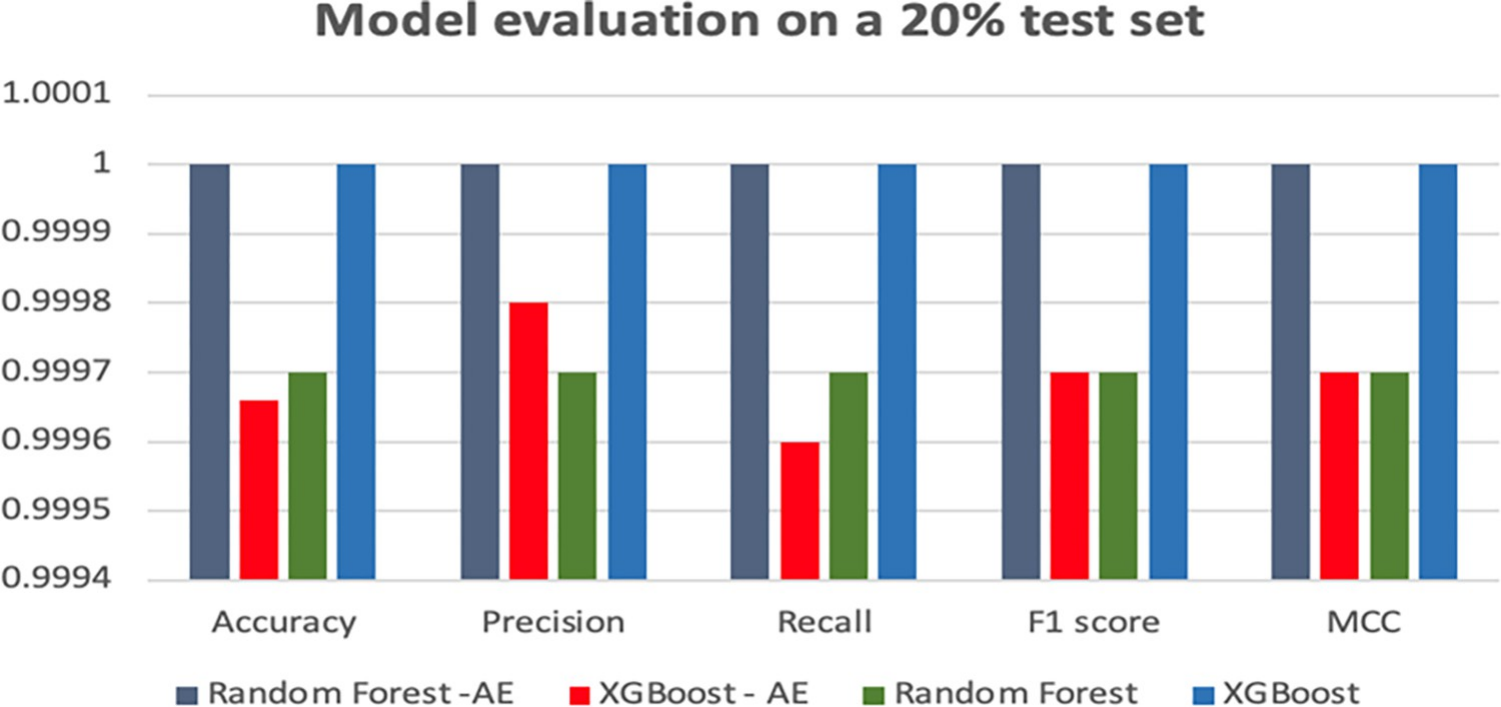
Model evaluation on a 20% test set.

Table 4 and Fig 13 depict the outcomes of the four models tested on 20% of the CIC-MalMem-2022 dataset.

**Table 4:** Model evaluation on a 20% test set.

Model	Accuracy	Precision	Recall	F1 score	MCC
Random Forest -AE	1	1	1	1	1
XGBoost—AE	0.99966	0.99980	0.99960	0.99970	0.9997
Random Forest	0.9997	0.9997	0.9997	0.9997	0.9997
XGBoost	1	1	1	1	1

Remarkably, both the XGBoost model and the Random Forest-AE model exhibited strong performance, achieving perfect scores for accuracy, precision, recall, F1 metrics, and MCC. However, these results suggest a potential issue of overfitting in the dataset. The XGBoost-AE and Random Forest models also garnered near-perfect scores. Upon observation, the XGBoost-AE model displayed slightly inferior performance compared to the other three models. Nevertheless, overall, it is evident that all four models performed admirably on the test set.

### Compare with previous research

**Fig 14:**
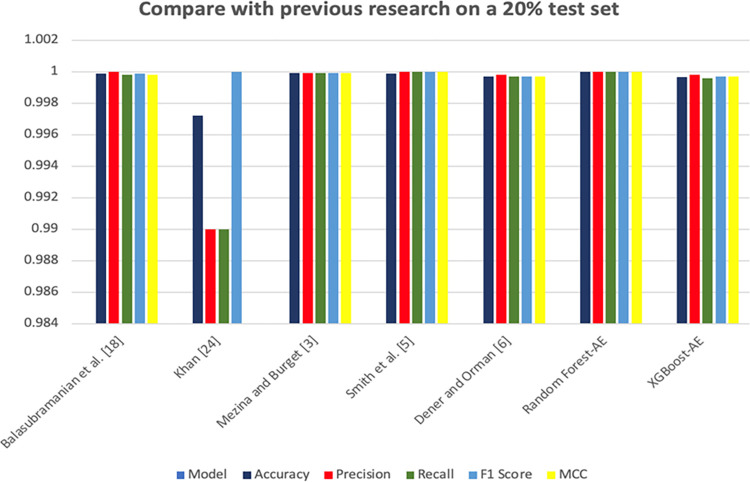
Previous research model evaluation on a 20% test set.

In contrast to previous studies, the present study introduces two new models and rigorously tests them by comparing them with the methods proposed in Mezina and Burget [3], Smith et al. [5], and Dener and Orman [6]. The rationale behind this comparative analysis is that these studies utilize the same dataset (i.e., CIC-MalMem-2022). Despite the differences in methodologies used in these studies, it is still reasonable to make comparisons between the models. Table 5 and Fig 14 illustrate the best-performing models from the reference study and the two models proposed in this study.

**Table 5:** Results compared with previous research.

Selected Related Work	Model	Accuracy	Precision	Recall	F1 Score	MCC
Balasubramanian et al. [18]	Random Forest	0.99990	1.00000	0.99980	0.99990	0.99980
Khan [24]	Artificial Neural Network	0.99720	0.99000	0.99000	1.00000	0.98260
Mezina and Burget [3]	Random Forest	0.99992	0.99992	0.99992	0.99992	0.99992
Smith et al. [5]	Decision Tree	0.99990	1.00000	1.00000	1.00000	1.00000
Dener and Orman [6]	Logistic Regression	0.99970	0.99980	0.99970	0.99970	0.99970
Propose by this research	Random Forest-AE	1.00000	1.00000	1.00000	1.00000	1.00000
Propose by this research	XGBoost-AE	0.99966	0.99980	0.99960	0.99970	0.99970

The evaluation metrics are based on the test set of the CIC-MalMem-2022 dataset split. Remarkably, all models achieve full or near-full scores on all metrics.

However, upon observation, it is noted that the performance of the model presented in this study (Random Forest-AE) achieves higher scores compared to those of the referenced models. This variance in performance could be attributed to the application of anomaly detectors, which enhance the models’ performance.

A thorough review of previous studies indicates that the CIC-MalMem-2022 dataset maintains a high fit to variables even after meticulous data pre-processing. One possible explanation for this finding is that the dataset creator did not sufficiently sample the data, resulting in an unrepresentative feature distribution. Another possibility is that the dataset creator may have designed effective features that led to near-perfect model performance when using this dataset as a basis. These findings underscore the need for further research into dataset features in the future.

#### Evaluate with unseen data

**Fig 15:**
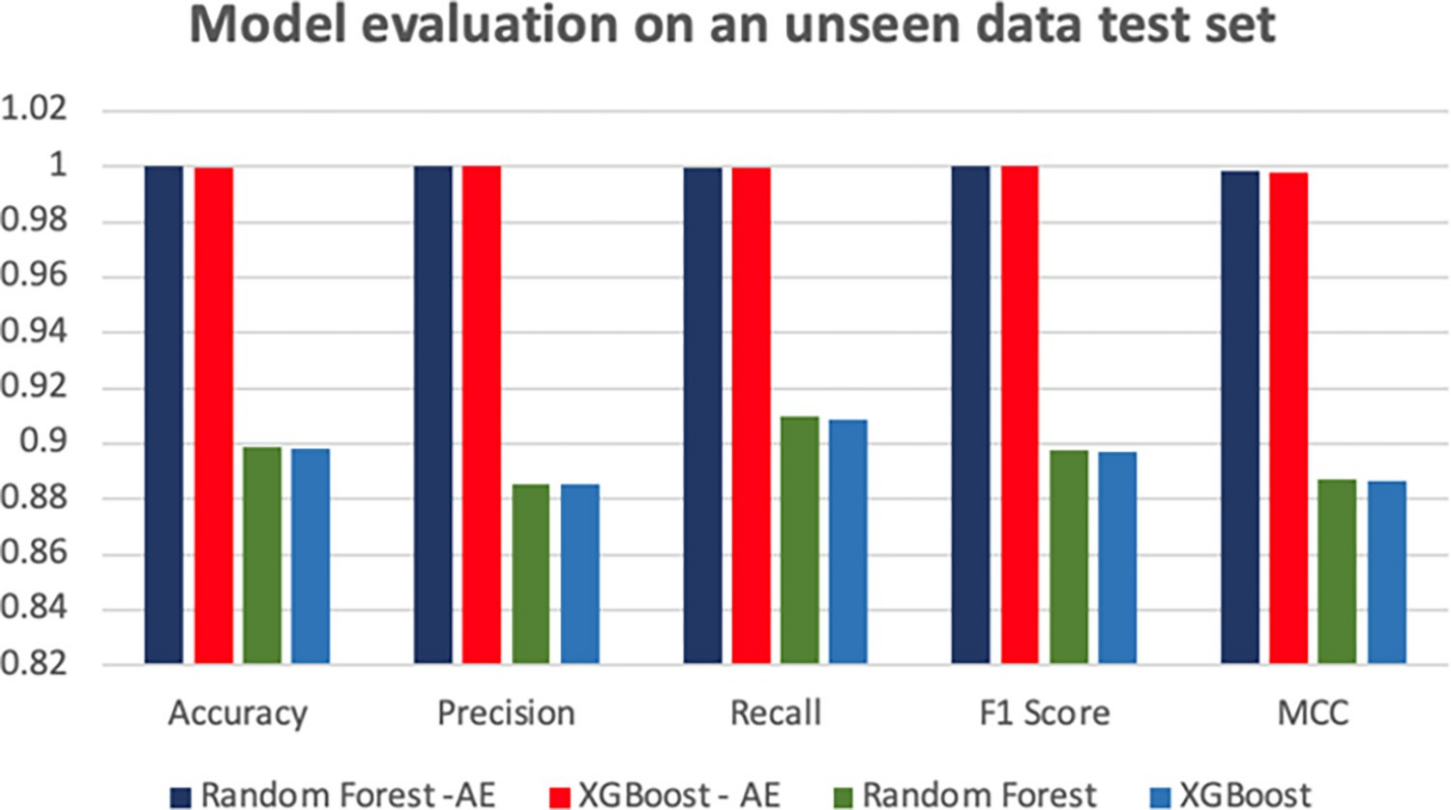
Model evaluation on an unseen data test set.

The combined performance evaluation results presented in Fig 15 and Table 6 focus on assessing the effectiveness of four models when applied to an unseen dataset. Specifically, the traditional Random Forest and XGBoost models, when used without the inclusion of the Autoencoder (AE) [25], exhibited a noticeable degradation in performance when detecting the unseen dataset.

**Table 6:** Model evaluation on an unseen test set.

Model	Accuracy	Precision	Recall	F1 Score	MCC
Random Forest -AE	0.999892	1.000000	0.999803	0.999901	0.998313
XGBoost—AE	0.999741	1.000000	0.999533	0.999976	0.998002
Random Forest	0.898837	0.885443	0.909734	0.897424	0.887162
XGBoost	0.898407	0.885344	0.908849	0.896943	0.886582

This decline in accuracy, precision, recall, F1 score, and MCC underscores the limitations of these models when faced with data outside their training domain.

Interestingly, the autoencoder-enhanced variants, namely Random Forest-AE and XGBoost-AE, demonstrated remarkable resilience in their performance metrics, with minimal changes in accuracy, precision, recall, F1 score, and MCC compared to their non-AE counterparts. This consistency in results suggests that the autoencoder’s ability to capture intrinsic features during training contributes to the robustness of the model, even when encountering previously unseen data.

### Insights and discussion

1. **Autoencoder Integration:** The integration of an autoencoder significantly improves the model’s ability to handle previously unseen data by learning the characteristics of normal traffic and identifying deviations. This step is critical to enhancing the model’s anomaly detection capability.
2. **Performance Consistency:** The autoencoder-enhanced models showed remarkable consistency across training, test, and unseen datasets. This consistency indicates robust generalization, which is essential for real-world applications where new types of attacks frequently emerge.
3. **Overfitting Concerns:** Although all models exhibited high performance, the near-perfect scores suggest a potential issue of overfitting, particularly with the CIC-MalMem-2022 dataset. The dataset’s high fit to the model variables could indicate that it may not fully represent real-world complexity.
4. **Comparative Advantage:** The Random Forest-AE model, in particular, outperformed methods proposed by Balasubramanian et al., Khan, Mezina et al., Smith et al., and Dener et al. This highlights the effectiveness of incorporating anomaly detection into traditional models.

## Challenges and solutions

Deploying the proposed models in real-world environments involves addressing several challenges related to computational costs, parameter sensitivity, and calibration. Below, we discuss these challenges and propose potential solutions.

### Computational costs

1. **Resource Intensity**: Training and deploying machine learning models, especially those incorporating autoencoders and ensemble methods like Random Forest and XGBoost, can be computationally intensive. The training phase requires significant computational power, particularly for large datasets like CIC-MalMem-2022. Real-time anomaly detection involves processing large volumes of data, necessitating robust infrastructure to handle the computational load.
2. **Scalability**: The models must scale efficiently to handle increased data volumes as network traffic grows. This requires optimizing the implementation to ensure that resource usage is kept within acceptable limits while maintaining performance.
3. **Potential Solutions**: Utilizing distributed computing frameworks such as Apache Spark can help manage large datasets and parallelize the computation, reducing the overall training and inference time. Leveraging hardware accelerators like GPUs or TPUs can significantly speed up the training and inference processes for deep learning models.

### Parameter sensitivity

1. **Hyperparameter Tuning**: The performance of machine learning models is highly dependent on the choice of hyperparameters. Parameters such as the number of trees in Random Forest, the learning rate in XGBoost, and the architecture of the autoencoder need careful tuning to achieve optimal performance.
2. **Regularization**: Regularization techniques (e.g., L1, L2 regularization) are essential to prevent overfitting, but finding the right balance is critical. Over-regularization can lead to underfitting, where the model fails to capture essential patterns in the data.
3. **Potential Solutions**: Tools such as grid search, random search, and Bayesian optimization can automate the process of hyperparameter tuning, helping to find optimal settings efficiently. Implementing cross-validation techniques can provide a more reliable estimate of model performance and stability across different parameter settings.

### Calibration

1. **Model Calibration**: Calibration is necessary to ensure that the model’s predicted probabilities reflect the true likelihood of an event. Poorly calibrated models can lead to overconfident predictions, which are particularly problematic in cybersecurity, where false negatives and false positives carry significant consequences.
2. **Confidence Intervals**: Providing confidence intervals for predictions helps in understanding the uncertainty associated with each prediction. This is crucial for making informed decisions in real-world scenarios where the cost of misclassification is high.
3. **Potential Solutions**: Techniques like Platt scaling and isotonic regression can be used to calibrate the predicted probabilities of machine learning models, improving their reliability. Using ensemble methods like stacking can help improve calibration by combining the strengths of multiple models.

### Implementation strategies

1. **Incremental Learning**: Deploying models that can learn incrementally from new data without requiring complete retraining from scratch. This approach helps in adapting to evolving threats in real-time and reduces the computational costs associated with full retraining.
2. **Monitoring and Maintenance**: Continuous monitoring of model performance in production is essential to detect and address any degradation in performance over time. Regular maintenance schedules, including periodic retraining with updated datasets, are necessary to keep the models effective against new attack vectors.
3. **Integration with Existing Systems**: Ensuring seamless integration with existing security infrastructure is critical. The models should be compatible with standard protocols and data formats used in current cybersecurity systems.

### Future work

To further validate the proposed models and address the limitations identified in this study, future research will involve evaluating the models on additional public datasets and implementing cross-dataset validation to provide insights into their generalizability across different types of cyber threats and data distributions. Additionally, exploring real-world deployment challenges such as computational costs, parameter sensitivity, and calibration will be crucial for ensuring the practical applicability and robustness of the models.

## Conclusion

This study presents a novel approach to detecting zero-day attacks by integrating autoencoders with traditional machine learning algorithms, specifically Random Forest and XGBoost. The proposed models, Random Forest-AE and XGBoost-AE, leverage the strengths of both anomaly detection and supervised learning to effectively identify previously unseen cyber threats. Using the CIC-MalMem-2022 dataset, the models were evaluated based on several metrics, demonstrating superior performance compared to existing methods.

The Random Forest-AE model achieved an accuracy of 99.9892%, precision of 100%, recall of 99.9803%, F1 score of 99.9901%, and MCC of 99.8313%. Similarly, the XGBoost-AE model achieved an accuracy of 99.9741%, precision of 100%, recall of 99.9533%, F1 score of 99.9976%, and MCC of 99.8002%. These results underscore the models’ robustness and ability to generalize to unseen data, outperforming baseline models that do not incorporate anomaly detection techniques.

Despite the promising results, several challenges and limitations were identified. The reliance on the CIC-MalMem-2022 dataset, while comprehensive, represents a specific snapshot of malware threats and may not capture the full diversity of cyber threats. Additionally, the computational costs associated with training and deploying the models are significant, requiring robust infrastructure and optimization strategies. The models are also sensitive to hyperparameter settings, necessitating careful tuning to achieve optimal performance. Finally, real-world deployment poses challenges such as integration with existing systems and continuous monitoring to maintain effectiveness over time.
